# *Citrus Yellow Vein Clearing Virus* Infection in Lemon Influences Host Preference of the Citrus Whitefly by Affecting the Host Metabolite Composition

**DOI:** 10.3390/plants14020288

**Published:** 2025-01-20

**Authors:** Yong-Duo Sun, Christopher M. Wallis, Rodrigo Krugner, Raymond Yokomi

**Affiliations:** United States Department of Agriculture, Agricultural Research Service, San Joaquin Valley Agricultural Sciences Center, Parlier, CA 93648, USA; christopher.wallis@usda.gov (C.M.W.); rodrigo.krugner@usda.gov (R.K.)

**Keywords:** citrus virus disease, insect vectors, citrus whitefly, spirea aphids, terpenes/terpenoids, sugars, amino acids, phenolics

## Abstract

Plant viruses have been known to alter host metabolites that influence the attraction of insect vectors. Our study investigated whether *Citrus yellow vein clearing virus* (CYVCV) infection influences vector attractiveness, focusing on the citrus whitefly, *Dialeurodes citri* (Ashmead). Free choice assays showed that citrus whiteflies exhibited a preference for settling on CYVCV-infected lemon plants versus healthy control plants. Using chromatography techniques, we found that the levels of sugars were similar in leaves and stems of both plant groups, while the contents of several amino acids in leaf or stem samples and non-volatile phenolic compounds in the leaf samples of CYVCV-infected and healthy plants differ drastically. In addition, volatile terpenes/terpenoids decreased significantly in virus-infected plants compared to healthy controls. Several of the identified volatile compounds such as α-phellandrene, α-terpinolene, p-cymene, linalool, and citral are known for their whitefly repellent properties. Further Y-tube olfactometer bioassays revealed that emissions of volatile organic compounds (VOCs) from infected plants attracted more citrus whiteflies, but not alate spirea aphids, *Aphis spiraecola* Patch, than those from healthy plants, suggesting that the VOCs released from CYVCV-infected lemon plants may specifically affect citrus whiteflies. Therefore, we suggest that, in addition to the visual cue of yellow vein symptoms, the preference of citrus whiteflies that settled on CYVCV-infected lemon plants was attributed to a reduction in the levels of repellent volatile compounds.

## 1. Introduction

Many invasive plant viruses are transmitted by insect vectors such as whiteflies and aphids [1] and pose an economic threat due to crop yield reductions [2]. Plant virus transmission by insect vectors involves a complex interplay between the pathogen, vector, and host plant. Plant infection by viruses can indirectly influence the behavior of insect vectors by affecting host plant physiology and phenotype, potentially impacting virus spread [3,4,5]. Virus-infected plants can be more attractive to vectors compared to healthy plants [6]. For example, *Bemisia tabaci* showed greater abundance on *Tomato yellow leaf curl virus*-infected plants compared to mock-inoculated plants [7]. Similarly, non-viruliferous apterous *Myzus persicae* preferred *Potato leaf roll virus* (PLRV)-infected potato plants over mock-inoculated plants [8]. The attractiveness of virus-bearing plants to insect vectors may be attributed to virus-induced alterations in visual phenotype, host immune responses, and metabolite compositions, including nutritional content and volatile organic compounds (VOCs) [9].

Changes in virus-induced VOCs can influence insect vector behavior as there are reports that aphid vectors use volatile chemical cues to locate hosts [10,11]. Host plants release various VOCs during virus infection, including terpenes, sesquiterpenes, green leaf volatiles, fatty acid derivatives, aromatics, nitrogen-containing compounds, and volatile plant hormones [12]. The composition and role of VOCs vary across different virus–insect and vector–host plant pathosystems. Several VOCs have been shown to play roles in the olfactory response of insect vectors, attracting them to virus-infected plants. For instance, VOCs induced by viruses such as *Cucumber mosaic virus* (CMV), *Barley yellow dwarf virus*, and PLRV attract specific non-viruliferous insect vectors to feed on virus-infected hosts [8,10,13]. Previous studies also found that insect vectors perform better on virus-infected host plants than non-infected plants due to lower volatile emissions compared to healthy plants [14,15,16,17]. For example, CMV and *Tomato yellow leaf curl China virus* inhibit the emission of terpenes that repel insect vectors [15,17].

In addition to volatile compounds, non-volatile compounds produced by host plants may also shift in response to viral and other non-fastidious pathogen infections, with potential consequences on insect vector feeding [6,18,19]. These compounds may include phenolic compounds, which are induced as a common response to both pathogen and insect feeding presumably to protect plants against biological attack and aid in symptom amelioration [20,21], as well as primary metabolites such as amino acids and sugars [18].

*Citrus yellow vein clearing virus* (CYVCV), a member of the virus subgenus *Mandarivirus*, genus *Potexvirus*, family Alphaflexiviridae, poses a significant threat to the citrus industry worldwide, affecting citrus-growing regions in Turkey, India, Iran, China, South Korea, and the United States [22,23,24,25]. CYVCV-infected citrus trees, such as lemon (*Citrus limon*) and sour orange (*C. aurantium*), exhibit stunted growth, reduced yields, yellow vein clearing, water-soaked vein appearance on the abaxial side, leaf deformities, intermittent ringspots, and venial necrosis [23,26,27]. The rapid spread of CYVCV in China since 2009 has led to substantial losses in lemon production [28]. In addition to being transmitted through grafting and mechanical practices, CYVCV has been reported to be naturally vectored by at least three aphid species (spirea aphid: *Aphis spiraecola*, cowpea aphid: *A. craccivora*, and cotton aphid: *A. gossypii*) and the citrus whitefly, *Dialeurodes citri* [29,30,31]. However, it remains unclear whether plant infection by CYVCV alters the host selection behavior of insect vectors.

In this study, using Eureka lemon, one of the most susceptible citrus varieties to CYVCV damage, and citrus whiteflies as models, we examined whether citrus whiteflies were more attracted to CYVCV-infected plants than healthy control plants. Further Y-tube olfactometry revealed that citrus whiteflies, but not spirea aphid, preferred CYVCV-infected lemon versus non-infected plants. Lastly, primary and secondary metabolites were observed in healthy and infected plants to potentially explain observations. Observations provide insight to the tri-partite interactions between CYVCV, its hosts, and its vectors, a better understanding of which may assist in developing novel strategies to control this virus disease for more sustainable and robust citrus production.

## 2. Results

### 2.1. Plant Infection by CYVCV Alters the Settling Behavior of Non-Viruliferous Citrus Whiteflies

**Figure 1:**
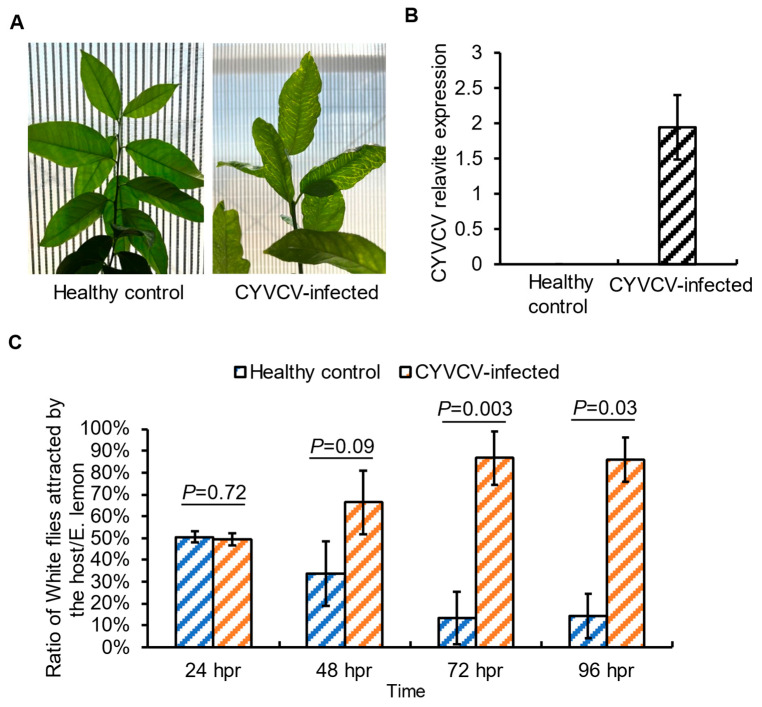
*Citrus yellow vein clearing virus* (CYVCV)-infected Eureka lemon attracted more citrus whiteflies compared to the healthy control. (**A**). A typical branch of Eureka lemon (*Citrus limon*) showing a typical CYVCV-induced yellow vein clearing phenotype and healthy control in the greenhouse. (**B**). RT-qPCR data verify the infection of CYVCV in the virus-bearing plants. The citrus Nad5 gene was used as an internal control. A similar trend was obtained from three biological repeats. (**C**). Periodic observation of the citrus whitefly free-choice bioassays. A similar trend was obtained from three biological repeats. hpr: hours post (insect)-release.

Free choice assays were set up to test whether the CYVCV infection could influence the settling behavior of citrus whiteflies. Phenotypically, the healthy control and CYVCV-inoculated lemon plants exhibit a similar growth condition. Yet, compared to the healthy control, the virus-infected lemon plants show characteristic symptoms, including yellow vein clearing and water-soaked vein appearance on the abaxial side. To ensure the virus infection, RT-qPCR was applied to quantify the virus titers in both plant sets (Figure 1A,B). The settling behaviors of non-viruliferous citrus whiteflies on CYVCV-infected lemon plants versus the healthy control were recorded over 96 h following insect release. There were no discernible settling preferences between the healthy and virus-infected plants during the initial 24 h of the choice test. However, by 48 h, whiteflies exhibited a settling preference for CYVCV-infected lemon plants over the control plants. This preference persisted throughout the remaining 72 h post-release (hpr). At 96 hpr, despite a decrease in the total number of citrus whiteflies, the proportion of whiteflies present on CYVCV-infected lemon plants continued to increase (Figure 1C). Three biological replicates yielded similar results (Table S1), indicating a consistent trend.

### 2.2. CYVCV Infection Modulates the Profiles of Amino Acids, but Not Sugars in Eureka Lemon

**Figure 2:**
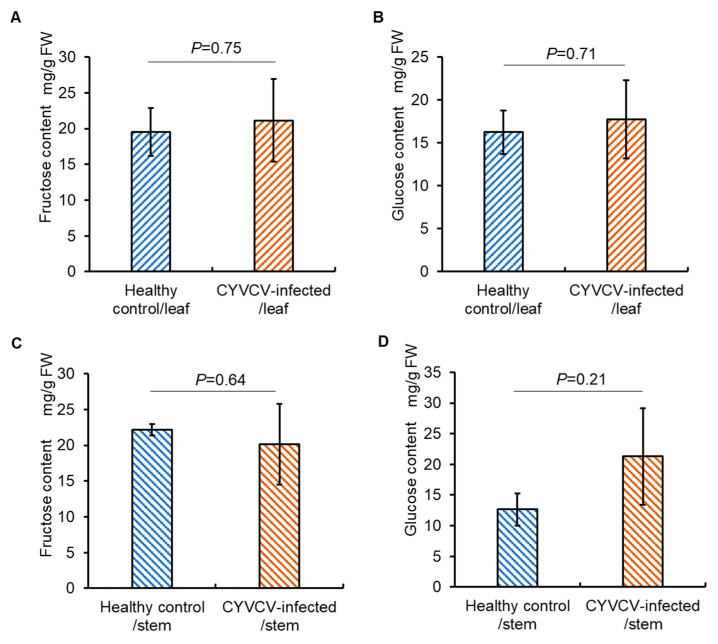
Sugar analysis in samples of *Citrus yellow vein clearing virus* (CYVCV)-infected Eureka lemon and healthy controls. (**A**,**B**). Fructose and Glucose content in the leaves of CYVCV-infected Eureka lemon and healthy plants. (**C**,**D**). Fructose and glucose content in the stems of CYVCV-infected Eureka lemon and healthy plants. Three biological repeats were conducted. No significant difference was observed in all measurements using the two-tail Student’s *t*-test. FW: fresh weight.

The attractiveness of CYVCV-infected plants to citrus whiteflies may be attributed to many virus-induced alterations such as nutritional change, visual and/or olfactory cues. Initially, we investigated whether CYVCV infection could impact plant nutritional content, particularly sugars and amino acids, potentially influencing whitefly preference. HPLC measurements revealed no significant differences in fructose and glucose content of leaves or stems between CYVCV-infected Eureka lemon plants and healthy controls (Figure 2A–D; Data S1). Thus, CYVCV infection did not affect the profile of primary metabolites fructose and glucose in the Eureka lemon.

**Figure 3:**
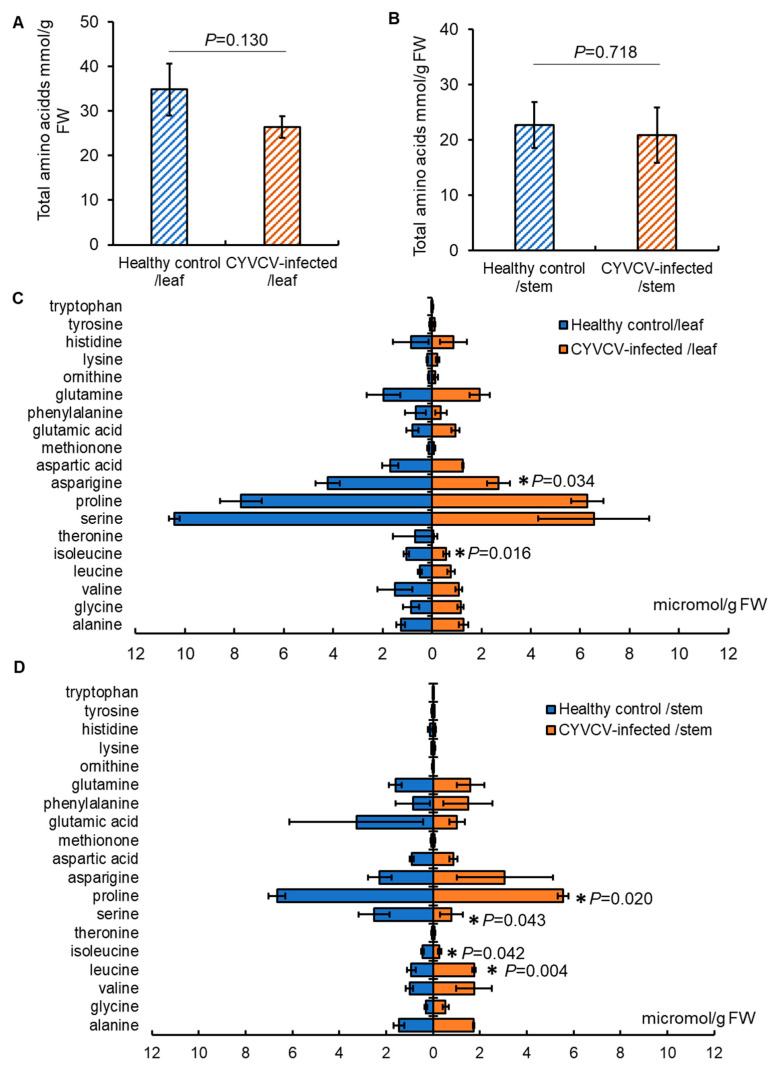
Amino acid analysis in samples of *Citrus yellow vein clearing virus* (CYVCV)-infected Eureka lemon and healthy control. (**A**), Content of total amino acids in the leaves of CYVCV-infected Eureka lemon and healthy control. (**B**), Content of total amino acids in the stems of CYVCV-infected Eureka lemon and healthy control. (**C**), Content of 19 individual amino acids in the leaves of CYVCV-infected Eureka lemon and healthy control. No significant difference was observed in any of the comparisons with *p* > 0.05 using the two-tail independent *t*-test. (**D**), Content of 19 individual amino acids in the stems of CYVCV-infected Eureka lemon and healthy control. Two-tail independent *t*-test. Three biological repeats were conducted. FW: fresh weight. *, *p* < 0.05.

Similarly, GC-MS analysis showed no significant differences in the total amino acid content of leaf and stem samples between CYVCV-infected Eureka lemon plants and healthy controls (*p* = 0.130 for leaf, *p* = 0.718 for stem; *t*-test) (Figure 3A,B; Data S1). The levels of 17 individual amino acids (alanine, glycine, valine, leucine, threonine, serine, proline, aspartic acid, methionine, glutamic acid, phenylalanine, glutamine, ornithine, lysine, histidine, tyrosine, tryptophan) between the two sets of plant leaf samples were similar, whereas the levels of isoleucine and asparagine (*p* = 0.016 and 0.034, respectively; *t*-test) decreased in the CYVCV-infected leaves compared to those in healthy controls (Figure 3C; Data S1). In stem samples, the changes in individual amino acids varied. The content of leucine increased (*p* = 0.004; *t*-test), whereas the content of isoleucine, serine, and proline (*p* = 0.042, 0.043, and 0.020, respectively; *t*-test) decreased drastically in the CYVCV-infected stem samples compared to healthy controls (Figure 3D, Data S1). These findings showed that CYVCV infection could modulate the profile of amino acids in Eureka lemon.

### 2.3. CYVCV Infection Alters the Content of Specific Soluble Phenolics in Eureka Lemon

**Figure 4:**
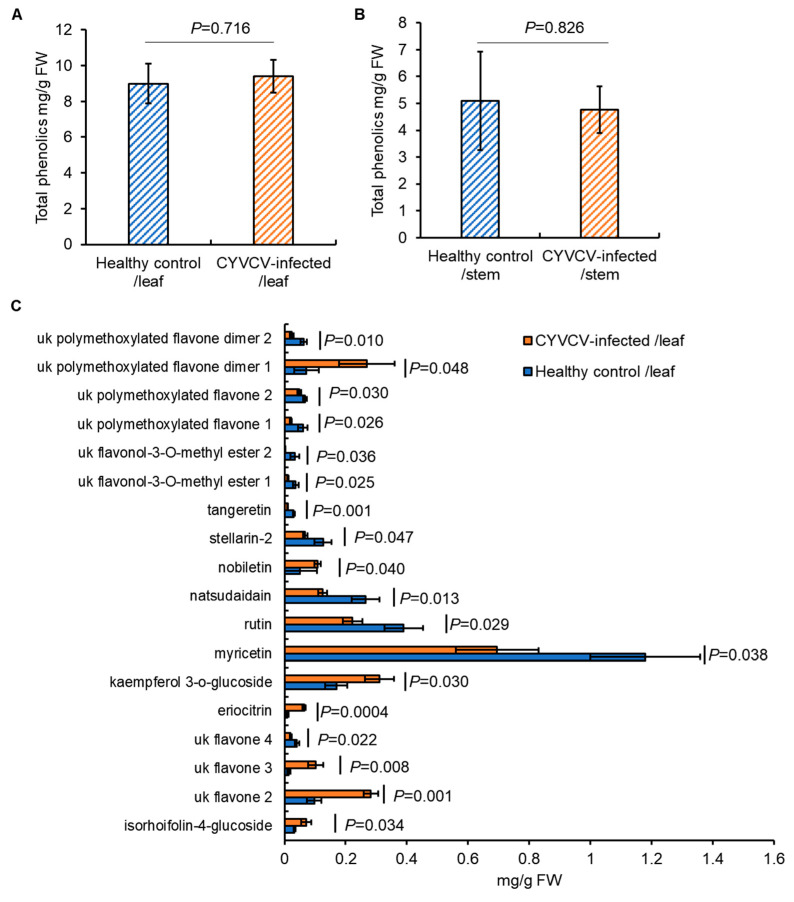
Phenolics levels detected in leaves of *Citrus yellow vein clearing virus* (CYVCV)-infected Eureka lemon, compared to those in healthy control. (**A**). Total phenolics levels in the leaves of healthy and CYVCV-infected Eureka lemon. No significant difference was observed with *p* > 0.05 using the two-tail Student’s *t*-test. (**B**). Total phenolics levels in the stem of healthy and CYVCV-infected Eureka lemon. Three biological repeats were conducted. No significant difference was observed with *p* > 0.05 using the two-tail Student’s *t*-test. (**C**). Eighteen individual phenolics that exhibit significant difference in the two groups of leaf samples. Three biological repeats were conducted. uk: unknown.

Subsequently, we examined phenolic compounds, a major group of biologically active secondary metabolites known to influence the orientation responses of hemipteran vectors. HPLC analysis detected 47 phenolic compounds (Data S2), with the total phenolic content remaining similar in leaves or stems between CYVCV-infected Eureka lemon plants and healthy controls (*p* = 0.716 for leaf sample, *p* = 0.826 for stem sample; *t*-test) (Figure 4A,B). By comparing individual compounds, there were no significant differences in levels of 47 compounds between the two sets of stem samples. However, the levels of 18 individual phenolic compounds in leaf samples differed due to CYVCV infection. Specifically, CYVCV infection resulted in significantly higher levels of seven phenolic compounds (i.e., isorhoifolin-4-glucosde, unknown flavone 2, unknown flavone 3, eriocitrin, kaempferol 3-o-glucoside, nobiletin, and unknown polymethoxylated flavone dimer 1 (*p* = 0.034, 0.001, 0.008, 0.0004, 0.030, 0.040, and 0.048, respectively, *t*-test)) than control plants (Figure 4C). Conversely, eleven phenolics, namely unknown flavone 4, myricetin, rutin, natsudaidain, stellarin-2, tangeretin, unknown flavonol-3-O-methyl ester 1, unknown flavonol-3-O-methyl ester 2, unknown polymethoxylated flavone 1, unknown polymethoxylated flavone 2, and unknown polymethoxylated flavone dimer 2 (*p* = 0.022, 0.038, 0.029, 0.013, 0.047, 0.001, 0.025, 0.036, 0.026, 0.030, and 0.010, respectively, *t*-test), were decreased in CYVCV-infected plants when compared to control plants (Figure 4C). Together, the profile changes of soluble phenolics in the leaves may influence the settling behavior of citrus whiteflies, though the roles of these phenolics in deterring or attracting whiteflies have not been conclusively demonstrated.

### 2.4. CYVCV Infection Influences the Metabolite Profiles of Volatile Terpenoid

**Figure 5:**
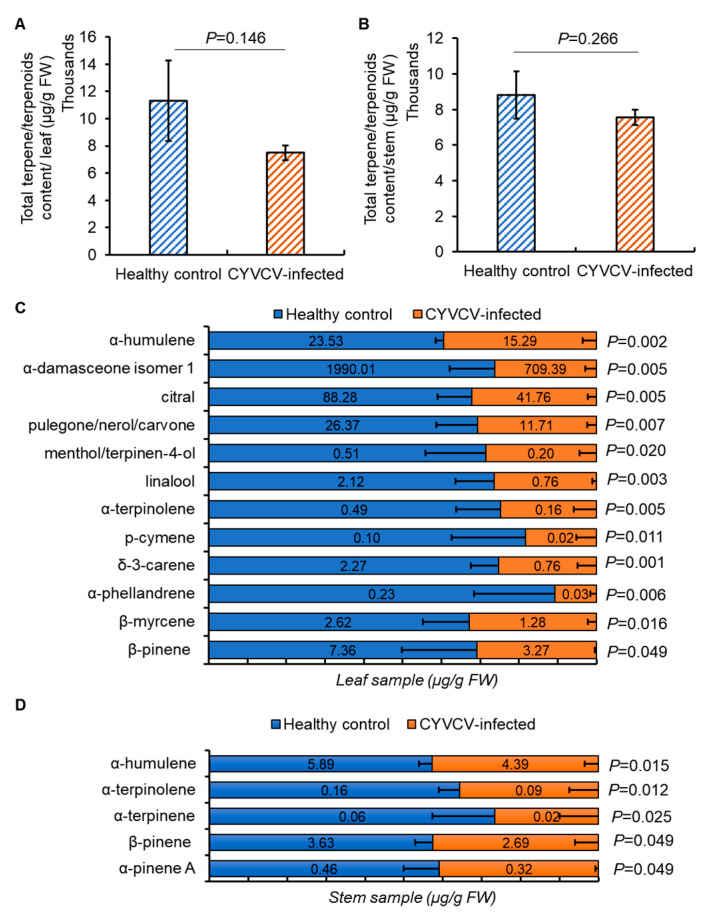
Volatile terpene/terpenoid analysis in samples of *Citrus yellow vein clearing virus* (CYVCV)-infected Eureka lemon and healthy control. (**A**). Total terpene/terpenoid levels in the leaves of CYVCV-infected Eureka lemon and healthy plants. No significant difference was observed. (**B**). Total terpene/terpenoid levels in the stem of CYVCV-infected Eureka lemon and healthy plants. Three biological repeats were conducted. No significant difference was observed. (**C**). Twelve individual terpenoids exhibit significant difference in the two groups of leaf samples. (**D**). Five individual terpenoids exhibit significant difference in the two groups of stem samples.

The Citrus genus is known to produce over 100 distinct VOCs, with terpenoids being the predominant group, which modulate the vector behaviors [32,33]. In the following study, we analyzed terpenoids extracted from leaf and stem samples of CYVCV-infected lemon and healthy control plants using GC-MS analyses. While total terpenoid levels in leaf and stem samples did not differ between the two sets of lemon plants (*p* = 0.146 for leaf sample, *p* = 0.266 for stem sample; *t*-test), the profiles of individual terpenoids exhibited notable alterations. Among the 29 terpenoids detected, the levels of 12 individual terpenoids (β-pinene, β-myrcene, α-phellandrene, δ-3-carene, p-cymene, α-terpinolene, linalool, menthol/terpinen-4-ol, pulegone/nerol/carvone, citral, α-damasceone isomer 1, and α-humulene) were at reduced levels (*p* ≤ 0.05) in CYVCV-infected leaf samples compared to healthy controls. In comparison, profiles of five individual terpenoids (α-pinene A, β-pinene, α-terpinene, α-terpinolene and α-humulene) were present at lowered levels in CYVCV-infected stem samples compared to healthy controls. Interestingly, none of the detected terpenoids occurred at greater levels in CYVCV-infected samples compared to healthy controls (Figure 5; Data S3).

Terpenoids displaying significant profile differences were primarily classified as monoterpene (α-pinene A, β-pinene, α-terpinene, β-myrcene, α-phellandrene, δ-3-carene, p-cymene, α-terpinolene, pulegone/nerol/carvone). The other major compound classes were monoterpenoids (terpinen-4-ol, linalool), monoterpene aldehyde (citral), ketones (α-damasceone isomer 1), and sesquiterpenes (α-humulene). Remarkably, many of these terpenoids, such as α-phellandrene [34,35], p-cymene [34,36], linalool [37], and citral [36,37], have been demonstrated to play roles in repelling or reducing host attractiveness to whiteflies.

### 2.5. Citrus Whiteflies Exhibit a Preference to the Air Current Carring Volatile Compounds Released from CYVCV-Infected Plants Other than the Healthy Control in Y-Tube Olfactometer Bioassays

**Figure 6:**
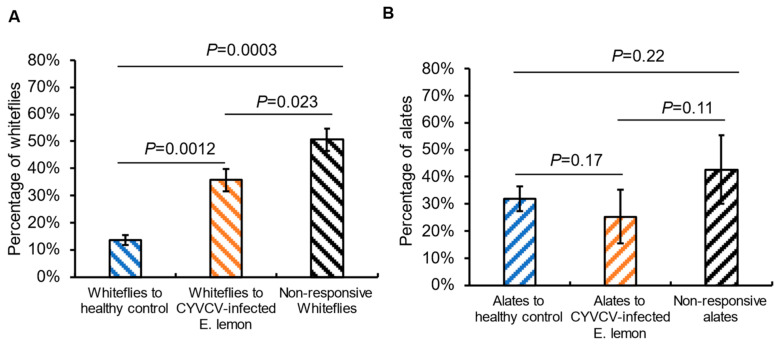
Vector response to volatile organic compounds released from *Citrus yellow vein clearing virus* (CYVCV)-infected Eureka lemon versus the healthy control in Y-tube olfactometry assays. (**A**). Percentage of whiteflies attracted by CYVCV-infected Eureka lemon and the healthy plants. (**B**), Percentage of spirea aphids attracted by CYVCV-infected Eureka lemon and the healthy plants. Statistical differences were analyzed using two-tail Student’s *t*-test.

In the next step, we examined whether VOCs released from infected plants contribute to attracting citrus whiteflies using the Y-tube olfactometer. In the olfactometry assays, citrus whiteflies responded to the VOCs and exhibited a preference to the air current carrying VOCs from CYVCV-infected plants. Although a notable proportion (approximately 50%) of citrus whiteflies did not exhibit a response within the 15 min test duration, among those that made a choice, there was a significant difference (*p* = 0.0012; *t*-test) in the number of responsive citrus whiteflies toward the VOCs from the infected plants. On average, about 40% of citrus whiteflies selected the arm connected to the CYVCV-infected plants in the Y-tube olfactometer, whereas approximately 15% of citrus whiteflies chose the arm representing healthy controls (Figure 6A, Table S2). Consequently, our findings suggest that VOCs emanating from CYVCV-infected host plants serve as cues influencing the host preferences of citrus whiteflies.

### 2.6. CYVCV-Induced VOCs Profile Changes in Lemon Tree Have Limited Effect on Olfactory Response of Spirea Aphids

For plant viruses with multiple vector species, even minor differences in the VOCs emitted by the host plant may elicit diverse effects on different insect vector species. This is because the insect host selection behavior is a complex process involving various blends of attraction or repellent semiochemicals [38]. Considering that CYVCV can also be transmitted by at least three aphid species, we investigated whether the VOCs emitted from CYVCV-infected plants also attract the aphid vectors. In subsequent Y-tube olfactometry assays, spirea aphid alates were used as models to assess their responses to the odors released from healthy control and CYVCV-infected plants. Similar to the observations with citrus whiteflies, a significant portion of the alate aphids did not exhibit a response within the 15 min observation period. However, no statistically significant difference was observed in the number of remaining aphids attracted to CYVCV-infected lemon plants compared to healthy controls (Figure 6B, Table S3). Thus, it appears that with spirea aphids and CYVCV, odor cues may not play a significant role in host selection.

## 3. Discussion

### 3.1. CYVCV Infection in Citrus Plant May Alter the Orientation Behavior of Citrus Whitefly

During the evolutionary trajectory of plant virus–insect vector–plant host interactions, a myriad of strategies has emerged to bolster virus vector transmission. This concept is encapsulated within the “Vector Manipulation Hypothesis” [39]. Although this hypothesis remains subject to scrutiny, numerous observations lend credence to its validity. For instance, in certain pathosystems, plant viruses directly affect vector behavior [40]. Another significant modulation occurs when viruses prompt alterations in the structural or chemical defenses of host plants, thereby impacting the settling and feeding behavior of insect vectors [10,41,42]. In this study, our findings indicate that CYVCV infection influences the orientation behaviors of its citrus whitefly vector, with virus-infected plants attracting more vectors compared to controls, though the efficiency of citrus whitefly in transmitting CYVCV California isolates remains unclear.

### 3.2. The Alternation of Citrus Non-Volatile Metabolites Profile Induced by CYVCV Infection Plant May Affect the Orientation Behavior of Citrus Whitefly

Herbivore host selection is a complex process influenced by various factors. While CYVCV-induced yellowing veins serve as visual cues, evidenced by the observation that citrus whiteflies could be attracted by yellow sticky trap (unpublished data), changes in host non-volatile metabolites also likely play a significant role in guiding the settling behavior of citrus whiteflies [18,20,21]. Our HPLC and GC-MS analysis revealed that CYVCV infection could modulate the profile of both primary metabolites, amino acids, and the secondary metabolites, soluble phenolic compounds. Though the total content of those measured compounds remains comparable in leaf or stem samples, notable alterations were observed in the levels of several individual amino acids, as well as non-volatile phenolic compounds in leaf samples. These changes in non-volatile metabolites could potentially influence the attractiveness of infected plants to citrus whiteflies. For instance, the flavonol myricetin demonstrated a decline following CYVCV infection in Eureka lemon, which contributes to inducing resistance against whiteflies in *Solanum pennellii* and *S. galapagense* [43]. The concise roles of these non-volatile metabolites in interfering with citrus–citrus whitefly interaction need further investigation.

### 3.3. VOCs Released from CYVCV-Infected Plants May Play a Role in Attracting Citrus Whiteflies

Manipulation of plant VOCs by certain plant viruses could influence the behavior of insect vectors. For example, volatiles emitted by potato plants infected with potato leaf roll virus have been shown to attract and retain the virus vector, *M. persicae* [44]. Similarly, CMV employs its 2b protein, a viral suppressor of host RNAi, to induce odor-dependent aphid attraction [17]. However, the composition of plant volatiles is complex, and simply targeting major compounds may not suffice to alter the behavior of some insect vectors. Various blends of semiochemicals and minor compounds can exert diverse effects on insect attraction or repellence [38]. Profiles of virus-induced plant volatiles have been extensively studied for certain plant viruses, such as luteoviruses [13,14,45]. In our research, lemon plants infected with CYVCV had reduced amounts of VOCs compared to healthy controls. Previous studies indicated that many insect vectors perform better on virus-infected host plants than non-infected plants, likely due to the reduced emission of VOCs with repellent properties [13,15,16,17].

Terpenoids represent a significant portion of the chemical compounds emitted by host plants, which typically act as repellents against insect vectors. For example, CMV and *Tomato yellow leaf curl China virus* inhibit the emission of terpenes that repel insect vectors from virus-infected plants, facilitating the feeding behavior of the vectors [15,17]. In our study, among the terpenoids significantly altered due to CYVCV infection, many play a role in repelling whiteflies in other crops. For instance, α-phellandrene [34,35], p-cymene [34,36], linalool [37], and citral [36,37] have all been shown to repel or reduce the attractiveness of hosts to respective whiteflies. Therefore, it is plausible that citrus whiteflies are oriented toward CYVCV-infected host plants due to a reduction in repellent compounds rather than an increase in attractants. However, further studies are necessary to identify the behavioral responses of vectors to individual and blends of compounds prominent in these VOC blends.

## 4. Materials and Methods

### 4.1. Plants, Virus, and Insects

Young lemon plants (*Citrus limon* L. Burm.f. cv. Eureka) (Sapindales: Rutaecae) propagated on *C. macrophylla* Wester (Sapindales: Rutaecae) rootstock and grown in Cone-tainer pots*^®^* (Ray Leach “super cell” air pruning cone-tainer) measuring 3.8 cm × 18.4 cm (width × height) were acquired from a local certified commercial citrus nursery when the plants were approximately six-month old and 30 cm tall above the budunion. Citrus plants from this nursery are produced from pathogen-free budwood and certified to be virus-free by the California Department of Food and Agriculture (CDFA), and the Citrus Clonal Protection Program at the University of California, Riverside, California. The lemon plants were boxed and transported to an evaporatively cooled BSL2 USDA-approved “protected structure” greenhouse in Parlier, California, under CDFA Permit No. QC 1581 and grown under pest- and virus-free conditions. The young lemon plants were transplanted to mini-treepots (10 cm × 14 cm) (width × height) in UC citrus soil mixture and maintained in the greenhouse with fertigation with Peters*^®^* Excel 21-5-20 Multi Purpose fertilizer (Everris NA Inc. Dublin, OH, USA) at 1% with water (10,000 ppm) and at a diurnal temperature of 24°/21° (day/night (d/n)) with supplemental high pressure sodium lighting during winter for a photoperiod of 16/8 d/n. The average annual daily greenhouse temperature ranged from 22° to 28 °C and the relative humidity ranged from 38 to 55%. The lemon plants for these experiments were moved to an air-conditioned quarantine greenhouse for virus inoculations under CDFA Permit No. 3926.

CYVCV was originally obtained from a fruit-bearing infected lemon tree in a dooryard in Tulare, California, and graft-propagated in the quarantine greenhouse in Parlier, California. The whole genome sequence was obtained by long-read sequencing technology with sequence data and annotation deposited in GenBank under the accession number OR037276.1 [27]. For this experiment, the Eureka lemon plants were approximately two years old with a stem diameter of 1 cm. The lemon plants were graft-inoculated with this CYVCV isolate from a symptomatic lemon plant in the greenhouse and a set of healthy controls were mock-inoculated with bark or leaf pieces from healthy lemon plants. Three months after graft inoculation, the infection statuses of the plants were assessed using reverse transcription real-time quantitative polymerase chain reaction (RT-qPCR) and used for the experiment.

The citrus whitefly, *Dialeurodes citri* (Ashmead) (Hempitera: Alyerodidae), was originally collected in 2023 from a field population infesting an organic mandarin (*C. reticulata* Blanco cv Tango) (Sapindales: Rutaecae) orchard near Reedley, California, and colonized in the insectary on *C. macrophylla*. The spirea aphid, *Aphis spiraecola* Patch (Hemiptera: Aphididae), was originally collected from an infestation in 2022 in a lemon orchard near Goleda, California and established on red-tip photinia (Photinia × fraseri) (Rosales: Rosaceae) plants in the insectary. The insectary in Parlier, California, was set like the greenhouse with a diurnal temperature of 24°/21° (d/n) and a photoperiod of 16/8 d/n. The average annual daily insectary temperature ranged from 21° to 24 °C and the relative humidity ranged from 43 to 53%. Adult citrus whiteflies and alate aphids were used for all experiments described below. Both the citrus whitefly and the spirea aphid are reported vectors of CYVCV [30,31].

### 4.2. CYVCV Detection by RT-qPCR

A duplex RT-qPCR method was employed for the simultaneous detection of CYVCV and the citrus Nad5 gene, serving as an internal quality control [27,46]. The RT-qPCR reaction was conducted in a 10 μL volume, comprising 2 μL of RNA template, 5 μL of 2× SuperScript III RT/Platinum Taq Mix reaction buffer (Invitrogen, Waltham, MA, USA), 0.4 μL each of CYVCV forward primer (5′-AAA TCC ATT AAC ACA GTG ACC TTC C-3′) and reverse primer (5′-AAC TCC TGA CAG TGC TCC AA-3′), along with 0.1 μM of a CYVCV-specific 6-FAM/BHQ-1 labeled TaqMan probe (5′d FAM-CGTCGTTGCCAAGACACGCCA-BHQ-1). Additionally, 0.4 μL each of Nad5 forward primer (5′-GATGCTTCTTGGGGCTTCTTKTT-3′) and reverse primer (5′-ACATAAATCGAGGGCTATGCGGATC-3′) were included, along with 0.1 μM of a Nad5-specific VIC/QSY labeled TaqMan probe (5′d VIC-CAT AAG TAG CTT GGT CCA TCT TTA TTCCAT-QSY). The reaction mixture was supplemented with 0.2 μL of iScript advanced reverse transcriptase (Invitrogen, Waltham, MA, USA) and 1 μL of double-distilled water. This blend was then distributed into a PCR plate, and the cycling conditions involved reverse transcription at 50 °C for 5 min and initial denaturation at 94 °C for 2 min, followed by 40 cycles of denaturation at 94 °C for 10 s, and annealing/extension at 60 °C for 40 s. RNA samples at a concentration of 10 ng/μL were tested in triplicate.

### 4.3. Free-Choice Bioassays

Free-choice experiments with citrus whiteflies were conducted in cages (Bug Dorms, MegaView Science Co., Ltd., Taichung, Taiwan) measuring 65 cm × 65 cm × 65 cm, maintained at a temperature of 25 °C, humidity of 65%, and a photoperiod of 16 h light and 8 h dark. In each experiment, three CYVCV-infected plants (6-month post inoculation) and three control plants were utilized. These plants were positioned in a circular arrangement inside the cage, equidistant from each other. Whiteflies, approximately 200 individuals in mixed sexes, were released from a Petri dish placed at the center of the circle. The number of whiteflies settling on each plant was then recorded at 24 h intervals over a period of 96 h to assess preferences over time. After the 96 h period, all plants hosting whiteflies were removed, and the cage was thoroughly cleaned before the next set of plants was arranged for the subsequential two biological repeats. Assays were mainly performed in September/October of 2023.

### 4.4. Y-Tube Olfactometer Bioassay

The Y-tube olfactometer bioassay was conducted following the methodology described by [47]. Briefly, the apparatus consisted of a pressure-regulated ultra-zero air tank (Westair, San Diego, CA, USA) set to deliver humidified air at a flow rate of 200 mL/min to a dual chamber olfactometer where chamber A held a CYVCV-infected plant (6 months post-inoculation) and chamber B held a CYVCV-free control plant. The Y-shaped tube had a 1.0 cm inner diameter with each arm being 8.0 cm length and at an angle of 60°. One arm of the Y-tube was connected to chamber A and the other to chamber B. Test insects were introduced at the single arm side and allowed to move freely toward the Y-tube bifurcation against the air current to choose between moving closer toward chamber A or chamber B. The bioassay was conducted in a climate-controlled room maintained at 25 °C temperature and 40–60% humidity. To minimize the influence of light, the Y-tube was positioned within a dark box.

A small cage containing a single citrus whitefly or spirea aphid was positioned at the base of the Y-tube, with equal amounts of air carrying VOCs from chamber A or chamber B to each arm of the Y-tube. The insect was released into the base of the Y-tube, where it was observed to move from downstream to upstream toward the air containing the VOCs from chamber A or chamber B. The insect’s movement across a marked line located 2.0 cm from the Y bifurcation within each branch was monitored. If the insect remained in its chosen branch for a period of 5 min (between two readings), it was considered to have made a choice. Any insect that did not make a choice within three consecutive readings (15 min) was recorded as a non-responder. A clean Y-tube was used for each new insect. In total, 123 spirea aphids (32, 30, 26, and 35, respectively, in four biological repeats) and 67 citrus whiteflies (20, 20, and 27, respectively, in three biological repeats) were exanimated. Assays were mainly performed in September/October of 2023.

### 4.5. Chemical Analyses

Amino acids, sugars, phenolic compounds, and terpenoid compounds were quantified following the procedures outlined by [48]. Amino acids were analyzed through a Shimadzu (Columbia, MD, USA) gas chromatograph–mass spectrometer (GC-MS)-QP2010s system equipped with a flame ionization detector (GC-FID). Briefly, a citrus leaf or stem from a healthy control or CYVCV-infected plant (6 months post-inoculation in September of 2023) was cryo-pulverized in liquid nitrogen using a mortar and pestle and subjected to solvent extraction using methanol (Thermo-Fisher, Pittsburgh, PA, USA). For each extraction, 100 mg of powdered tissue underwent sequential dual extraction with 500 µL of methanol solvent each at 4 °C overnight in a 1.5 mL microcentrifuge tube, resulting in a total of 1 mL of methanol. Next, amino acids on 100 µL of the methanol extract were proceeded with the Phenomenex (Torrance, CA, USA) EZ-FAAST free (Physiological) amino acid analysis kit and the subsequent solvent was injected to the GC-MS system for analysis via the Phenomenex ZB-AAA GC Column.

A Shimadzu (Columbia, MD, USA) LC-10AD pump-based high-performance liquid chromatography (HPLC) system was used to analyze sugars in methanol extraction, as mentioned above in amino acid analysis. Briefly, 50 µL of the methanol extract was injected into the HPLC system, which was equipped with a Supelcogel H column (25 cm × 4.8 mm × 9 µm) and a Shimadzu RID-10 Refractive Index Detector. The mobile phase, 0.1 M phosphoric acid (Thermo Fisher Scientific, Waltham, MA, USA), was delivered at a flow rate of 0.1 mL/min for 60 min per sample. Standard curves for sucrose and glucose were obtained to quantify the respective sugars in the sample.

Phenolic compound analysis was performed using a Shimadzu (Columbia, MD, USA) LC-20AD HPLC system equipped with a Supelco Ascentis RP-18 reverse-phase column (Sigma-Aldrich, St. Louis, MO, USA) and an SPD-20 photodiode array detector. Citrus samples were extracted with methanol, and 50 µL aliquots were analyzed. A binary gradient solvent, starting from 95% solvent A (water with 0.2% (*v*/*v*) acetic acid) and 5% solvent B (methanol with 0.2% acetic acid) to 100% solvent B and back over, was employed to separate the phenolic compounds. Phenolic compounds were detected at 280 nm and identified by comparing their UV/Vis spectra and retention times to commercial standards or published data. Quantification was achieved using standard curves of relevant phenolic compounds.

Terpenoid compound analysis was performed using a Shimadzu (Columbia, MD, USA) QP2010S GC-MS system equipped with a SHRXI-5MS column. Citrus samples were extracted with methyl tert-butyl ether (MTBE) containing 0.1% n-pentadecane as an internal standard. A 100 mg sample was subjected to two consecutive extractions with 500 µL MTBE at 4 °C overnight. Two microliters of the MTBE extract were injected into the GC-MS system with helium as the carrier gas. The oven temperature was programmed to increase from 60 °C to 200 °C for 35 min, and then to 250 °C for 8 min and 20 s, followed by a hold at 250 °C for 6 min and 40 s. Terpenoids were identified and quantified by comparison with commercial standards.

### 4.6. Statistic Analyses

All the statistical calculations and analyses conducted in the study were carried out using the “R” programming language. A two-tailed student *t*-test was used to check for significant differences between the two groups. Following a pair of comparisons during chemical analysis, a *t* value > 2.776 or *t* value < −2.776 (df = 4) with a *p* value ≤ 0.05 was treated as a significant difference. Only *p* values were denoted in figures.

## 5. Conclusions

This study reveals that CYVCV infection profoundly alters the metabolic landscape of lemon plants. Notably, the levels of some non-volatile metabolites, such as amino acids and phenolics, as well as some volatile terpenes/terpenoids, known for their insect-repellent properties, were modulated by CYVCV infection. This metabolic shift may have a direct impact on the settling behavior of virus vectors. Further investigation reveals that citrus whiteflies attracted to the modified VOC profile, exhibit a strong preference for CYVCV-infected plants over healthy controls. In contrast, spirea aphids show no such preference. Our findings suggest that, in addition to virus-induced visual cues, alteration of volatile emissions plays a crucial role in determining the host preferences of citrus whiteflies in lemon trees.

## Data Availability

Data are contained within the article and Supplementary Materials.

## Supplementary Materials

The following supporting information can be downloaded at https://www.mdpi.com/article/10.3390/plants14020288/s1: Data S1: Raw data of sugar and amino acid analysis in samples of *Citrus yellow vein clearing virus* (CYVCV)-infected and healthy Eureka lemon; Data S2: Raw data of phenolic compound measurements in samples of *Citrus yellow vein clearing virus* (CYVCV)-infected and healthy Eureka lemon; Data S3: Raw data of volatile terpene/terpenoid analysis in samples of *Citrus yellow vein clearing virus* (CYVCV)-infected and healthy Eureka lemon. Table S1: Timelapse calculation of citrus whitefly population attracted by CYVCV-infected Eureka lemon and the control plants in free-choice bioassays; Table S2: Statistical calculation of citrus whiteflies attracted by *Citrus yellow vein clearing virus* (CYVCV)-infected Eureka lemon and the healthy control plants in Y-tube olfactometry assays; Table S3: Statistical calculation of spirea aphid alates attracted by *Citrus yellow vein clearing virus* (CYVCV)-infected Eureka lemon and the healthy control plants in Y-tube olfactometry assays.
