# Supplementary material for: Citrus Yellow Vein Clearing Virus Infection in Lemon Influences Host Preference of the Citrus Whitefly by Affecting the Host Metabolite Composition
Source: Plants (Basel). 2025 Jan 20;14(2):288. doi: 10.3390/plants14020288 (PMC11768271; doi:10.3390/plants14020288)
Supplement: Supplementary file 1 [file plants-14-00288-s001.zip › Supplemental Tables 01.08.25.pptx]

## Slide 1
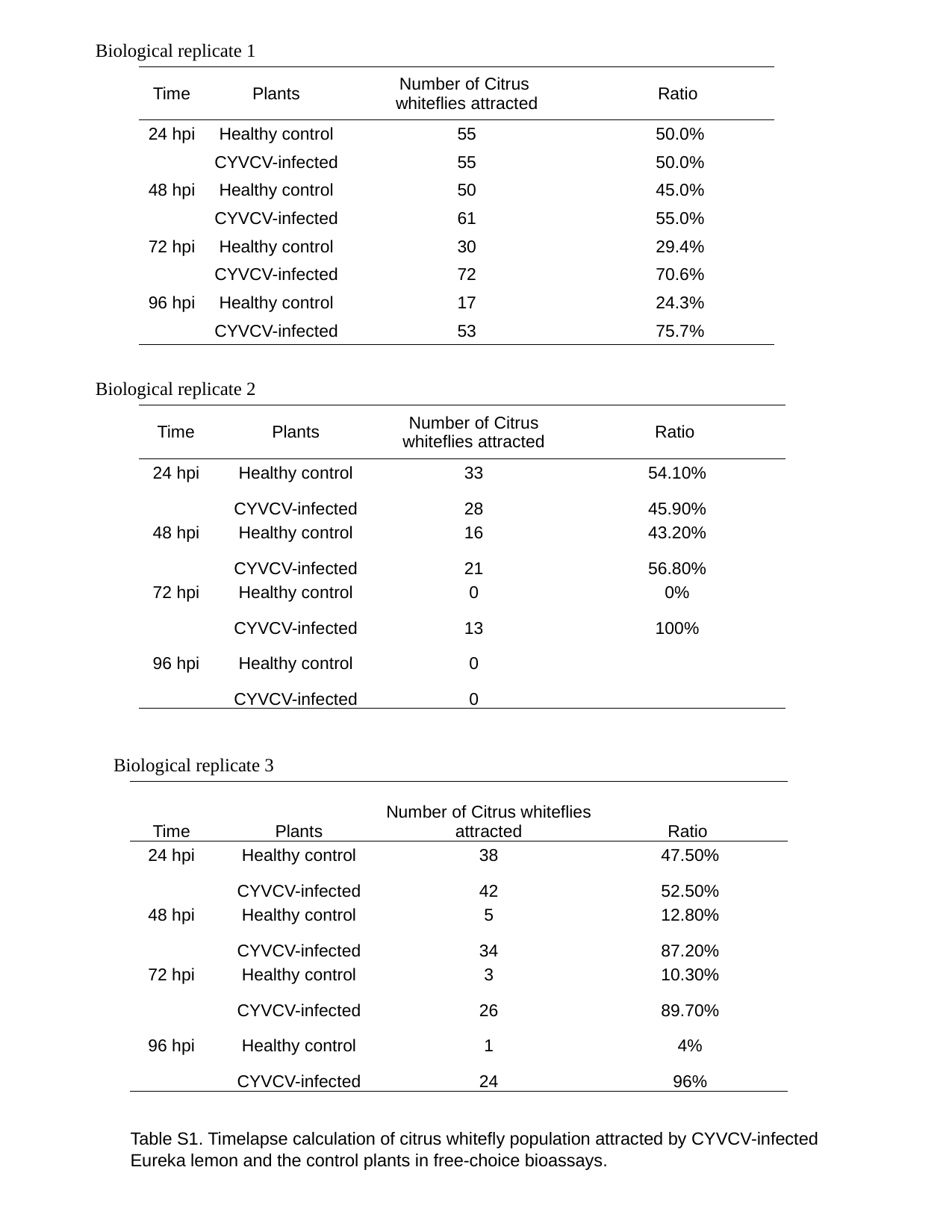

Biological replicate 1
| Time | Plants | Number of Citrus whiteflies attracted | Ratio |
| --- | --- | --- | --- |
| 24 hpi | Healthy control | 55 | 50.0% |
| | CYVCV-infected | 55 | 50.0% |
| 48 hpi | Healthy control | 50 | 45.0% |
| | CYVCV-infected | 61 | 55.0% |
| 72 hpi | Healthy control | 30 | 29.4% |
| | CYVCV-infected | 72 | 70.6% |
| 96 hpi | Healthy control | 17 | 24.3% |
| | CYVCV-infected | 53 | 75.7% |
Biological replicate 2
| Time | Plants | Number of Citrus whiteflies attracted | Ratio |
| --- | --- | --- | --- |
| 24 hpi | Healthy control | 33 | 54.10% |
| | CYVCV-infected | 28 | 45.90% |
| 48 hpi | Healthy control | 16 | 43.20% |
| | CYVCV-infected | 21 | 56.80% |
| 72 hpi | Healthy control | 0 | 0% |
| | CYVCV-infected | 13 | 100% |
| 96 hpi | Healthy control | 0 | |
| | CYVCV-infected | 0 | |
Biological replicate 3
| Time | Plants | Number of Citrus whiteflies attracted | Ratio |
| --- | --- | --- | --- |
| 24 hpi | Healthy control | 38 | 47.50% |
| | CYVCV-infected | 42 | 52.50% |
| 48 hpi | Healthy control | 5 | 12.80% |
| | CYVCV-infected | 34 | 87.20% |
| 72 hpi | Healthy control | 3 | 10.30% |
| | CYVCV-infected | 26 | 89.70% |
| 96 hpi | Healthy control | 1 | 4% |
| | CYVCV-infected | 24 | 96% |
Table S1. Timelapse calculation of citrus whitefly population attracted by CYVCV-infected Eureka lemon and the control plants in free-choice bioassays.

## Slide 2
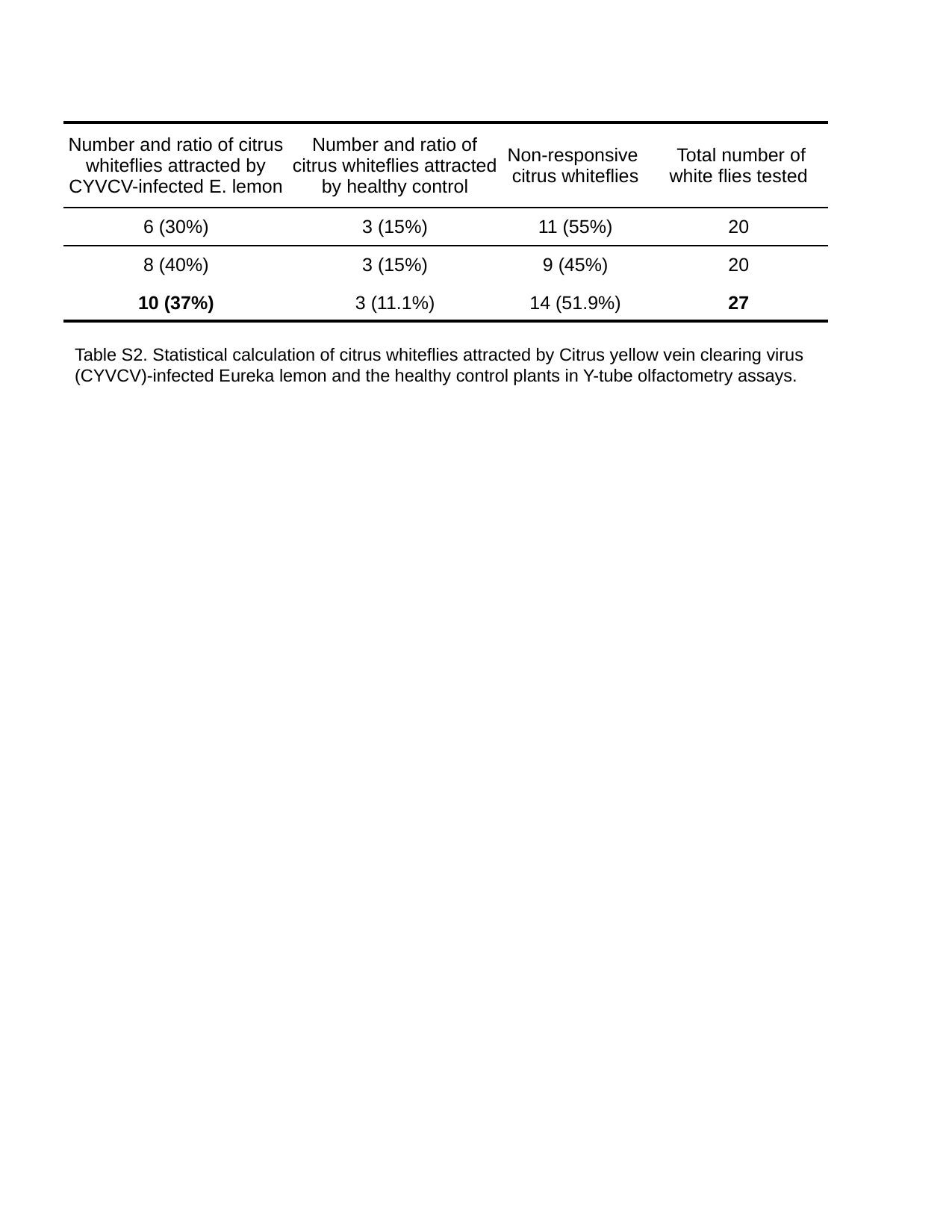

| Number and ratio of citrus whiteflies attracted by CYVCV-infected E. lemon | Number and ratio of citrus whiteflies attracted by healthy control | Non-responsive citrus whiteflies | Total number of white flies tested |
| --- | --- | --- | --- |
| 6 (30%) | 3 (15%) | 11 (55%) | 20 |
| 8 (40%) | 3 (15%) | 9 (45%) | 20 |
| 10 (37%) | 3 (11.1%) | 14 (51.9%) | 27 |
Table S2. Statistical calculation of citrus whiteflies attracted by Citrus yellow vein clearing virus (CYVCV)-infected Eureka lemon and the healthy control plants in Y-tube olfactometry assays.

## Slide 3
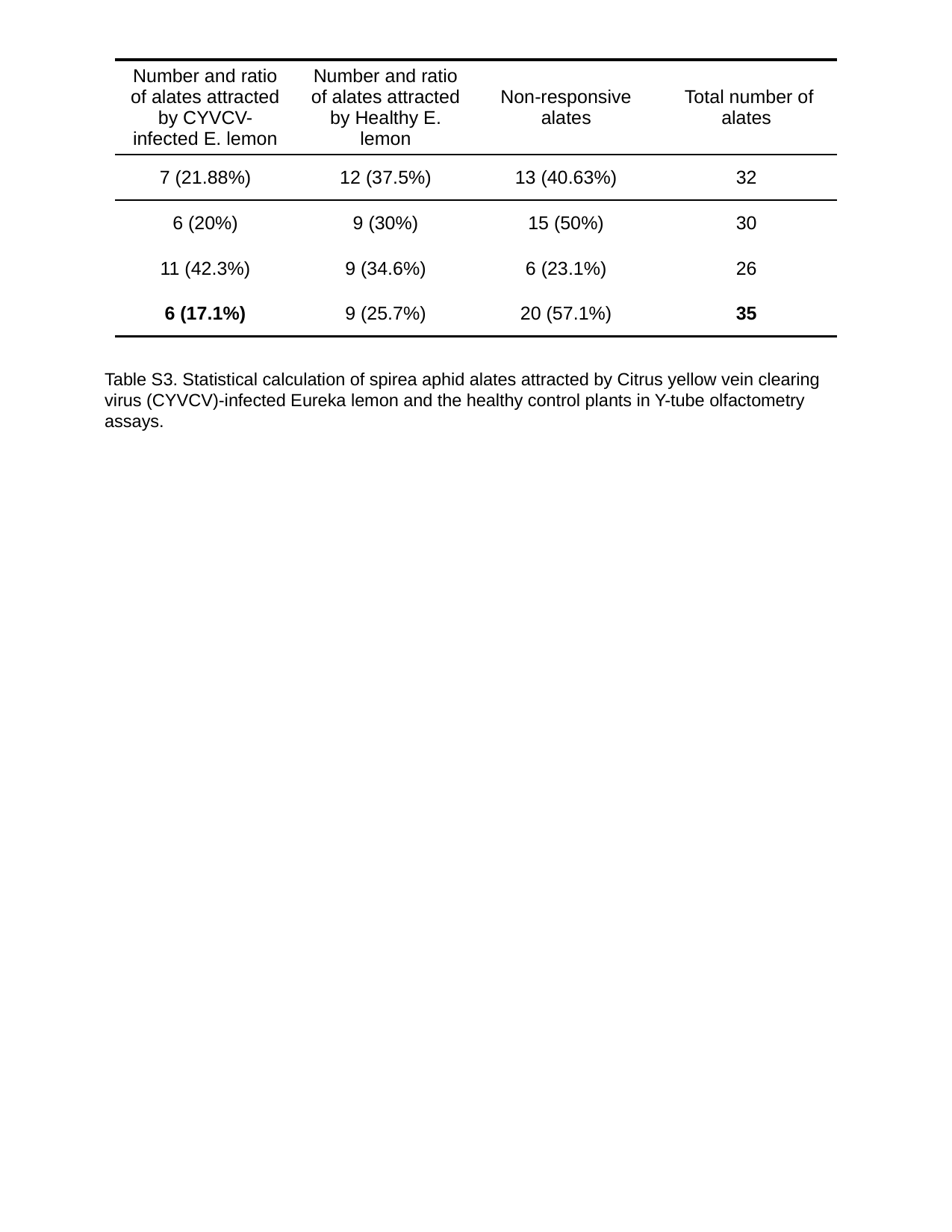

| Number and ratio of alates attracted by CYVCV-infected E. lemon | Number and ratio of alates attracted by Healthy E. lemon | Non-responsive alates | Total number of alates |
| --- | --- | --- | --- |
| 7 (21.88%) | 12 (37.5%) | 13 (40.63%) | 32 |
| 6 (20%) | 9 (30%) | 15 (50%) | 30 |
| 11 (42.3%) | 9 (34.6%) | 6 (23.1%) | 26 |
| 6 (17.1%) | 9 (25.7%) | 20 (57.1%) | 35 |
Table S3. Statistical calculation of spirea aphid alates attracted by Citrus yellow vein clearing virus (CYVCV)-infected Eureka lemon and the healthy control plants in Y-tube olfactometry assays.
